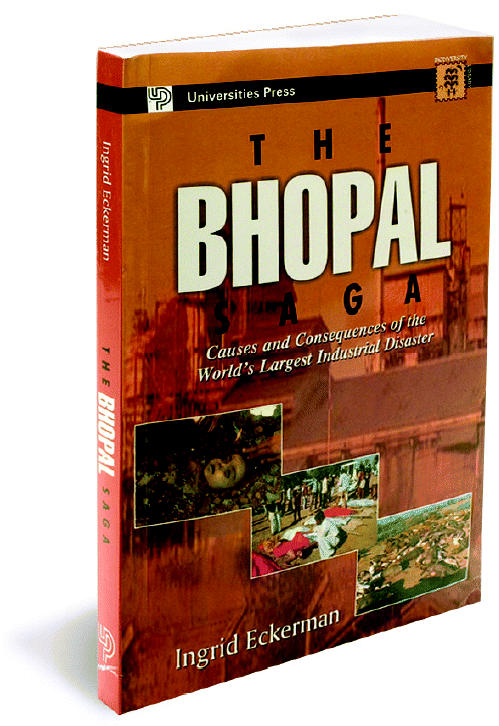# The Bhopal Saga: Causes and Consequences of the World’s Largest Industrial Disaster

**Published:** 2005-05

**Authors:** Carol S. Wood

**Affiliations:** Carol S. Wood is a toxicologist in the Life Sciences Division at Oak Ridge National Laboratory, Oak Ridge, Tennessee. She wrote the technical support documents on MIC and toluene diisocyanate for the National Advisory Committee on Acute Exposure Guideline Levels, which have been published by the National Academy Press. She also analyzes developmental, reproductive, and neurotoxicity data on a wide variety of pesticides.

By Ingrid Eckerman

Hyderabad, India:Universities Press, 2005. 283 pp. ISBN: 81-7371-515-7. Rs250

After 20 years, victims, health care workers, and governments are still trying to comprehend what has been called the world’s worst industrial accident. *The Bhopal Saga* is an attempt to bring order from the chaos of events before, during, and after the methyl isocyanate (MIC) release. Not a scientific analysis, the book summarizes events leading up to the accident of December 1984 and the relief work in the ensuing two decades.

Eckerman’s primary strength is her on-scene experience as a member of the International Medical Commission on Bhopal. She describes comprehensively the long-term health effects documented in the exposed population and suggests what might be done to improve health care for the victims. The paucity of data on certain end points, notably women’s reproductive health, childhood outcome, and cancer, is stressed. Also included is a summary of the positive and negative effects of various interim relief efforts on the population. Eckerman includes societal, economic, environmental, and political aspects that she considers imperative for integrated long-term health care.

The range of acute and chronic health effects in the exposed population is placed in terms of the toxicity of the probable major components of the gas cloud. Unfortunately, Eckerman relied on secondary sources for this information, although the database for most of the individual chemicals is robust and contains controlled experiments in both humans and animals. The primary literature would have informed her that beyond contact irritation, toxicity of the isocyanates varies widely. For example, MIC is not a sensitizer and causes systemic toxicity, whereas other isocyanates are potent sensitizers and local irritants only.

Nevertheless, the acute symptoms, delayed pulmonary effects, and chronic health problems are otherwise well correlated in the context of the known toxicities of the chemicals discussed. Both Union Carbide Corporation and the state and national Indian governments may deserve the constant accusations aimed at them in *The Bhopal Saga*, but this is almost a distraction. The known safety violations at the plant, the lack of education and training of workers, and the documented incidents of noncompliance before the leak speak for themselves; Eckerman even refers to the accident as a “massacre” or the “killings” in several places. The belief that Union Carbide is withholding data, especially the composition of the gas cloud, is speculation on her part. Current efforts by researchers in the United States to recreate the chemical reaction may soon answer the question of component gases. Although the issue is still debated in the medical community, Eckerman firmly believes that sodium thiosulfate should have been widely used as an antidote for the exposed victims: Eckerman concludes that sodium thiosulfate must have been necessary, not because of patient presentation, but because Union Carbide withdrew the initial recommendation for use of the cyanide antidote. Although it is probable that hydrogen cyanide was generated in the chemical reaction and was present in the gas cloud, cyanide has not been shown to be a breakdown product of MIC in human or animal systems. It is impossible to judge whether Union Carbide was basing its treatment recommendation on toxicity data or for some other reason.

A list of other accidents at Union Carbide plants worldwide contains a mistake (Table 10, p. 267). Large quantities of mercury were used in the 1950s and 1960s at a U.S. Department of Energy facility that was managed at the time by Union Carbide’s Nuclear Division. Eckerman incorrectly states that half of the workers involved were killed, when in fact no adverse health effects have been found. In addition, effects of released mercury on the environment and surrounding communities have been studied extensively.

Eckerman’s biases against industry and government can be forgiven when compensation was delayed for years, a reliable health care infrastructure is still not in place, environmental laws are not enforced, and worker safety appears compromised by lapses in oversight. Unfortunately, Eckerman offers no new solutions to the human rights issues specific to the Bhopal tragedy or to the world population in general.

## Figures and Tables

**Figure:**